# Measurement of different types of intelligence (general, verbal vs. non-verbal, multiple), academic performance and study habits of secondary students at a Music Integrated Centre

**DOI:** 10.1016/j.dib.2019.104124

**Published:** 2019-06-10

**Authors:** Amelia Barrientos-Fernández, Roberto Sánchez-Cabrero, Amaya Arigita-García, Lidia Mañoso-Pacheco, Francisco Javier Pericacho-Gómez, Miguel Ángel Novillo-López

**Affiliations:** a Department of Social Sciences and Applied Languages, Alfonso X el Sabio University, Madrid, Spain; b Department of Languages and Education, Nebrija University, Madrid, Spain; c Department of Geography and History, National Distance Education University (UNED), Madrid, Spain

**Keywords:** Musical intelligence, Multiple intelligences, Academic performance, Factorial intelligence, Music integrated centres

## Abstract

According to many relevant scientific researches conducted in the last few years [1–3], the study of music-related subjects implies greater development of both intellectual and executive functions of students. Those functions comprise musical intelligence [4] and the rest of multiple intelligences [5], as well as the general and factorial intelligence [6]. The present research may help students organise and plan their studies with an improvement of their study habits, thus better self-organising their daily work. Unfortunately, the percentage of secondary students at Music Integrated Centres is less than 0.01% of the total centres; indeed, there are only 10 centres in Spain out of 34,149 total number of non-university education centres of this type [7]. Hence, the sample obtained from this data collection is a *rara avis* of a great scientific value [8,9].

In this study, a sample of 28 first-year secondary students at a Music Integrated Centre has been collected. In Music Integrated Centres, learners simultaneously study the General Education System and music-related courses from their third year of Primary Education until the second year of Baccalaureate. In order to obtain the data, several measurement tests have been conducted, namely general and factorial intelligence, multiple intelligence and study habits. Moreover, the study collects the academic performance of students in two evaluations carried out the school year 2017–2018 of the general courses on Spanish Language (mother tongue of the students), Mathematics, Social Sciences, Natural Sciences and English as a Foreign Language, as well as the music-related subjects, in particular Musical Language, Instrument and Choir. The data gathered for this field study can be useful if related to other researches on students belonging to other levels and modalities at Secondary Education with a focus on multiple intelligences and learning strategies, among others.

**Table:** Table of specifications

Subject area	Education and Psychology
More specific subject area	General and factorial intelligence, multiple intelligences, academic performance, study habits, music-related studies
Data type	Tables, graphics and text file
Data collection method	Individual application of the following tests: General and Factorial Intelligence Test (IGF-5R, Yuste-Hernanz 2018) [10] IHE Questionnaire on techniques and study habits (Fernández-Pozar, 2014) [11] CUIM Questionnaire on Multiple Intelligences (Aliaga et al., 2014) [12] Academic performance of core and music-related subjects in the first year of Compulsory Secondary Education
Data format	Raw, unfiltered data
Experimental factors	No data pre-treatment of the selected sample
Experimental features	Firstly, the CUIM and IGF-5R questionnaires were applied, followed by the IHE questionnaire on study habits. Finally, the course tutor was requested the grades reflecting the academic performance of students considering the core and music-related subjects of the two evaluations of the first year of Compulsory Secondary Education
Data source location	Music Integrated Centre, Madrid, Spain
Data accessibility	The study data are available in the article
Related research article	M.C. Reyes-Belmonte, El rendimiento académico de los alumnos de primaria que cursan estudios artístico-musicales en la Comunidad Valenciana, Universitat de València, 2011. http://roderic.uv.es/handle/10550/25132

**Table:** 

**Value of the data** In this studio, the following must be highlighted: • It is an incidental sampling very difficult to localise due to the lack of students enrolled in this education system. Therefore, the sample is regarded as *rara avis*, a very valuable scientific asset. • The data can be useful to compare students coming from different levels, areas, studies and education centres with different characteristics. • The collected data are valuable to assess the influence the musical development has on certain aspects, such as general intelligence, factorial intelligence, multiple intelligences and the association among these factors. • The data are suitable for those studies aimed at justifying the incorporation of musical knowledge in the official curriculum. • The data can be used to test the influence the choice of the musical instrument type (wind, string or percussion) has on both general and musical academic performance.

## 1 Data

Tests were given to 28 students during two sessions. In the first one, CUIM [12] questionnaire on multiple intelligences, which measures the linguistic, musical, logic-mathematical, spatial, interpersonal, intrapersonal, coenesthetic, and naturalist intelligence, was given. In this session, the IGF-5R test on General and Factorial Intelligence [10] assessing verbal and non-verbal intelligence was also applied. In the second session, a questionnaire on study habits was employed [11]. Finally, the group tutor was requested the grades of students corresponding to their academic performance of the two evaluations of the first year of Compulsory Secondary Education.

The participants did not receive any previous treatment. Data are presented in.csv format. The information was originally processed on paper and then transformed into digital form by the statistical software SPSS in.sav format, which allowed a correct setting of variables for its later analysis.

The analysed variables are the following:

• General, verbal and non-verbal intelligence (quantitative variables measured on scale).
• Multiple intelligences (quantitative variable measured on scale).
• Study habits (quantitative variable measured on scale).
• Academic performance of core and music-related subjects (quantitative ordinal variable).
• Types of music instruments played (nominal variable).

Data have not been treated (raw data), which have been collected through the corresponding questionnaires and the students' own grades. The variables and their corresponding data are presented in a.csv file organised in columns in the following way:

• Raw score (PD) and percentile (PC) of:
  ○ General Intelligence (IG): measured on scale.
  ○ Non-verbal Intelligence (INV): measured on scale.
  ○ Verbal Intelligence (IV): measured on scale.
• Raw Score (PD) and DECA (DC) of:
  ○ Study habit environment: measured on scale.
  ○ Study habit planning: measured on scale.
  ○ Study habit materials: measured on scale.
  ○ Study habit assimilation: measured on scale.
• Direct scores of academic performance based on two evaluations (1 and 2) in the following subjects:
  ○ Natural Sciences 1 and 2 (scale); Fail (value 0)-pass (value 1) Natural Sciences (ordinal).
  ○ Social Sciences 1 and 2 (scale); Fail (value 0)-pass (value 1) Social Sciences (ordinal).
  ○ Spanish Language 1 and 2 (scale); Fail (value 0)-pass (value 1) Spanish Language (ordinal).
  ○ English as a Foreign Language 1 and 2 (scale); Fail (value 0)-pass (value 1) English as a Foreign Language (ordinal).
  ○ Mathematics 1 and 2 (scale); Fail (value 0)-pass (value 1) Mathematics (ordinal).
  ○ Average of students' academic performance in core subjects.
  ○ Musical Instrument 1 and 2; Fail (value 0)-pass (value 1) Musical Instrument.
  ○ Musical Choir 1 and 2; Fail (value 0)-pass (value 1) Musical Choir.
  ○ Musical Language 1 and 2; Fail (value 0)-pass (value 1) Musical Language.
  ○ Orchestra/band/group 1 and 2; Fail (value 0)-pass (value 1) Orchestra/band/group
  ○ Average of academic performance in music-related subjects (scale).
• Multiple intelligences:
  ○ Verbal-linguistic (scale).
  ○ Logical-mathematical (scale).
  ○ Spatial (scale).
  ○ Bodily-kinaesthetic (scale).
  ○ Musical (scale).
  ○ Intrapersonal (scale).
  ○ Interpersonal (scale).
• Types of music instruments played by students (categories):
  1. String (nominal).
  2. Wind (nominal).
  3. Percussion (nominal).

**Fig. 1:**
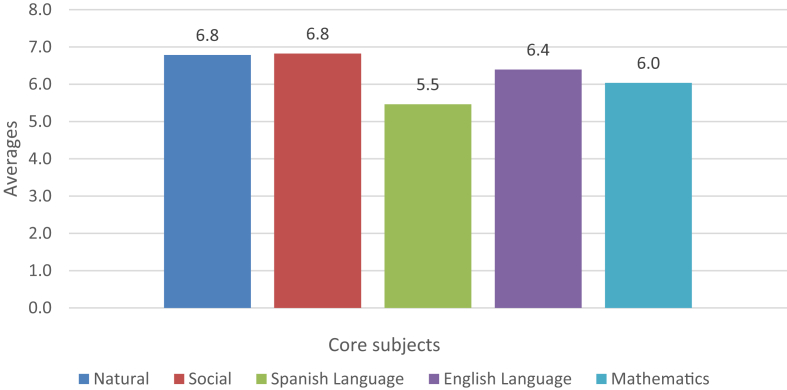
Core subjects' performance.

**Fig. 2:**
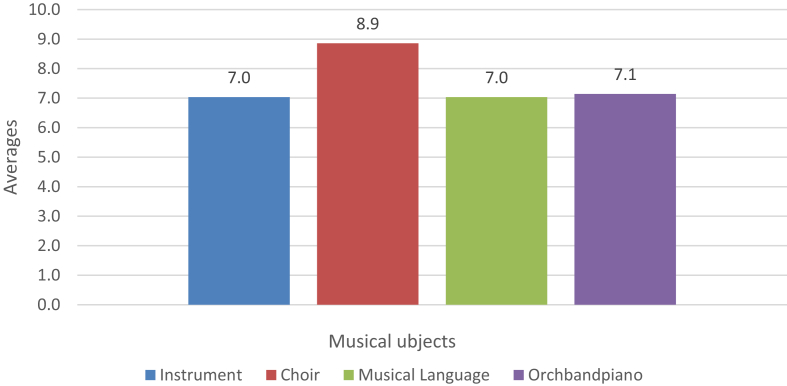
Musical subjects' performance.

**Fig. 3:**
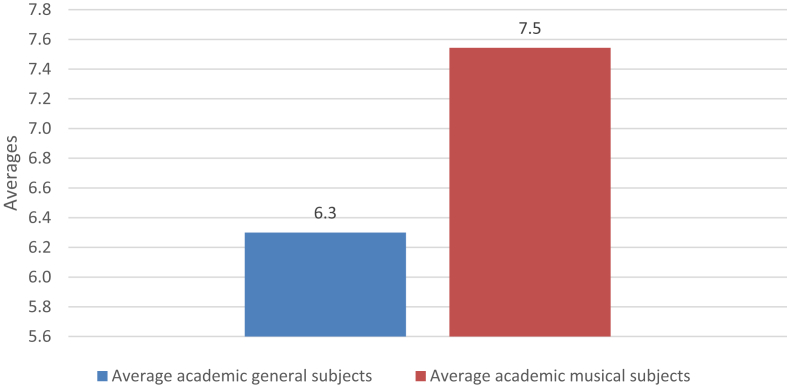
Comparison between general and musical averages.

**Fig. 4:**
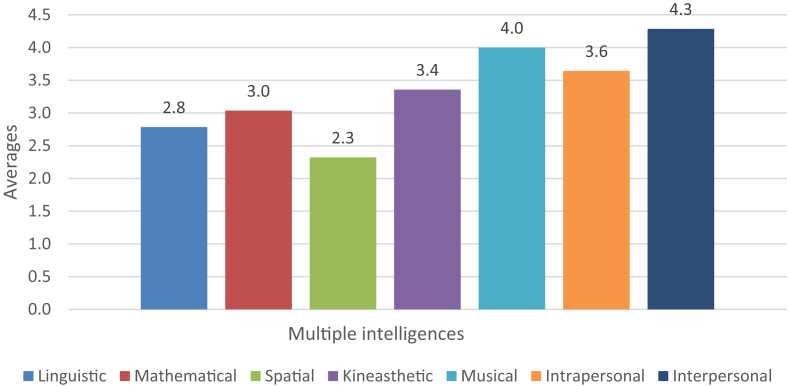
Multiple intelligences' averages.

The study presents the average of the academic performance in each of the core and music-related subjects (see Fig. 1, Fig. 2 for further information). On the other hand, the average of the core and music-related subjects are attached globally (see Fig. 3 for further information). Finally, the research shows the averages of the multiple intelligences (see Fig. 4 for further information).

## 2 Experimental design, materials, and methods

The population under study just comprises 10 out of the 34,149 total centres at Compulsory Secondary Education stage in Spain. In those centres, both core and music-related subjects were taught, as seen in Table 1 below [1], [2], [3].

**Table 1:** Music integrated centres in Spain.

Centre	Location	Type
Escolania de Lluc	Islas Baleares	Private
Sant Josep Obrer II	Islas Baleares	Private
Àgora Portals	Islas Baleares	Private
Unión Musical de Liria	Valencia	Private
El Drac	Valencia	Private
Simó Ballester	Islas Baleares	Public
Son Serra	Islas Baleares	Public
Oriol Martorell	Barcelona	Public
Federico Moreno Torroba	Madrid	Public
Padre Antonio Soler	Madrid	Public

In Music Integrated Centres, the ratio of students per class is 50% less than the rest of education centres belonging to secondary stage. Moreover, students must pass a placement test that assesses their musical knowledge. Then, the number of students per centre is approximately 25% with respect to the average figure in other secondary centres [7]. Thus, it is an incidental and specialised sampling, very sparse in the Spanish education field [4], [5], [6].

In order to get the sample of the study, one of the centres specified in Table 1 was visited, carrying out a cluster sampling. One of the public centres was chosen so that the socio-economic factors of the families of the students did not distort the selected sampling.

Considering the small population under study, as well as the observed broad spread (the Music Integrated Centres are only present in 4 major cities of the Spanish state), a single cluster may be regarded as representative of the whole population, since it means approximately a 10% of the total [8], [9].

Data collection was conducted in two distinct moments, visiting in person the Music Integrated Centre. The participants handwrote the questionnaires individually. The data were later digitalised for further statistical analysis via SPSS and Microsoft Office Excel.

In order to select the tests for the study, scientific tools were employed, which comply with the following conditions:

• Validated and assessed tests addressed to preadolescent and adolescent living in Spain.
• High internal reliability (measured by Cronbach's Alpha).
• Updated tests carried out in the last 5 years.
• Tests allowing for disaggregation by various sub-measures suitable for statistical analysis. Tests must also show direct and typical measurement (suitable for comparison with other tests, sampling and population).
• Tests allowing for application on paper and in an individual form.
• Tests that can be replicated with a length less than 30 minutes.

The tests used, complying with the above-described conditions, are as follows:

1. General, verbal and non-verbal intelligence questionnaire following IGF-5R General and Factorial Intelligence test (2018) [10], which consists of 70 items and Its execution time are 30 minutes. This test was first designed in 1991 and has received numerous updates since then. The test has high internal reliability (*Cronbach's Alpha*: 0.719).
2. Multiple intelligences following CUIM questionnaire on Multiple Intelligences (Aliaga et al., 2014) [12], which consists of 80 items at the Likert-type scale. It is executed in 15 minutes approximately and shows high internal reliability (*Cronbach's Alpha: 0.543*). It assesses the following:
  a. Linguistic intelligence.
  b. Logical-mathematical intelligence.
  c. Spatial intelligence.
  d. Musical intelligence.
  e. Bodily-kinaesthetic intelligence.
  f. Interpersonal intelligence.
  g. Intrapersonal intelligence.
3. Fernández-Pozar's Questionnaire on techniques and study habits (IHE) (2014) [11], which comprises 90 items and it is executed in 15 minutes. The test was first designed in 1981 and has received numerous updates since then. It shows high internal reliability (*Cronbach's Alpha: 0.766*) and assesses the following: work and study habits of students through these scales:
• Environmental conditions when studying.
• Study planning.
• Use of materials.
• Assimilation of contents.
• Sincerity (additional scale).

The research has been carried out from a descriptive approach. To that aim, statistical analysis of frequencies has been conducted and data have been detailed through the analysis of correlations among variables, as seen in Table 2 below. In particular, the comparison between the general and factorial intelligence indices, and the multiple intelligences degree; the correlation between general and musical performances, and study habits; and finally, the relation between general and musical performance, and the musical instruments chosen by the students.

**Table 2:** Descriptive data analysis.

Statistical descriptions	N	Minimum	Maximum	Average	Standard deviation
General intelligence	28	25	56	44.07	7.845
Non-verbal intelligence	28	7	35	22.36	6.361
Verbal intelligence	28	11	33	17.86	5.421
Study habit environment	28	5	30	22.79	5.789
Study habit planning	28	1	23	13.93	4.682
Study habit materials	28	4	24	16.96	5.725
Study habit assimilation	28	10	28	21.32	4.563
Study habit sincerity	28	6	25	18.36	4.002
Natural Sciences (1^st^ evaluation)	28	3	9	5.68	1.679
Natural Sciences (2^nd^ evaluation)	28	3	9	6.79	1.572
Social Sciences (1^st^ evaluation)	28	3	9	6.89	1.912
Social Sciences (2^nd^ evaluation)	28	3	9	6.82	1.847
Spanish Language (1^st^ evaluation)	28	3	8	5.32	1.467
Spanish Language (2^nd^ evaluation)	28	2	8	5.46	1.732
English as a Foreign Language (1^st^ evaluation)	28	3	9	6.11	1.988
English as a Foreign Language (2^nd^ evaluation)	28	3	10	6.39	2.079
Mathematics (1^st^ evaluation)	28	5	9	6.46	1.401
Mathematics (2^nd^ evaluation)	28	4	9	6.04	1.710
Average grades of core subjects	28	3.8	8.6	6.300	1.5611
Musical Instrument (1^st^ evaluation)	28	4	9	7.00	1.333
Musical Instrument (2^nd^ evaluation)	28	4	9	7.04	1.551
Musical Choir (1^st^ evaluation)	28	7	10	8.54	1.036
Musical Choir (2^nd^ evaluation)	28	6	10	8.86	1.145
Musical language (1^st^ evaluation)	28	5	10	7.46	1.453
Musical language (2^nd^ evaluation)	28	4	10	7.04	1.895
Orchestra/band/group (1^st^ evaluation)	28	4	9	6.61	1.449
Orchestra/band/group (2^nd^ evaluation)	28	6	9	7.14	.932
Average grades of musical subjects	28	5.8	9.0	7.543	.9527
Verbal-linguistic intelligence	28	1	5	2.79	1.101
Logical-mathematical intelligence	28	1	5	3.04	1.374
Spatial intelligence	28	1	5	2.32	1.219
Bodily-kinaesthetic intelligence	28	1	5	3.36	.989
Musical intelligence	28	1	5	4.00	1.186
Intrapersonal intelligence	28	1	5	3.64	1.062
Interpersonal intelligence	28	1	5	4.29	.976
